# Changes in hepatic haemodynamics in rats with overt liver tumour.

**DOI:** 10.1038/bjc.1991.469

**Published:** 1991-12

**Authors:** D. M. Nott, S. J. Grime, J. Yates, J. N. Baxter, T. G. Cooke, S. A. Jenkins

**Affiliations:** University Department of Surgery, Royal Liverpool Hospital, UK.

## Abstract

Overt liver tumour was induced in Fisher rats by intraportal administration of 1.6 x 10(7) Walker carcinosarcoma cells. Control groups of rats received similar volumes of dead cells or saline intraportally. All animals were studied at 3 weeks when overt tumour was present. The Hepatic Perfusion Index (HPI) was significantly raised in rats with overt tumour compared to both groups of control animals. Portal flow and portal venous inflow were significantly reduced in the presence of overt tumour but hepatic arterial flow did not alter. These observations suggest that the alteration in the HPI in the presence of overt tumour results from an alteration in portal venous flow and inflow even though the blood supply to the tumour is principally derived from the hepatic artery. The changes in hepatic haemodynamics in the presence of tumour were accompanied by a reduction in portal pressure, an increase in splanchnic vascular resistance and an increase in the degree of arteriovenous shunting through the liver. Portal vascular resistance was unchanged. These findings indicate that the presence of overt hepatic tumour results in gross derangements of hepatic blood flow. These changes must be taken into consideration when attempting to potentiate the delivery of cytotoxic drugs to hepatic tumour by manipulation of hepatic haemodynamics.


Br. J. Cancer (1991), 64, 1088–1092 © Macmillan Press Ltd., 1991

### Changes in hepatic haemodynamics in rats with overt liver tumour

D.M. Nott[1], S.J. Grime[2], J. Yates[1], J.N. Baxter[1], T.G. Cooke[1,*], & S.A. Jenkins[1]

*University Departments of [1]Surgery and [2]Nuclear Medicine, Royal Liverpool Hospital, Liverpool, UK.*

**Summary** Overt liver tumour was induced in Fisher rats by intraportal administration of $1.6 \times 10^7$ Walker carcinosarcoma cells. Control groups of rats received similar volumes of dead cells or saline intraportally. All animals were studied at 3 weeks when overt tumour was present. The Hepatic Perfusion Index (HPI) was significantly raised in rats with overt tumour compared to both groups of control animals. Portal flow and portal venous inflow were significantly reduced in the presence of overt tumour but hepatic arterial flow did not alter. These observations suggest that the alteration in the HPI in the presence of overt tumour results from an alteration in portal venous flow and inflow even though the blood supply to the tumour is principally derived from the hepatic artery. The changes in hepatic haemodynamics in the presence of tumour were accompanied by a reduction in portal pressure, an increase in splanchnic vascular resistance and an increase in the degree of arteriovenous shunting through the liver. Portal vascular resistance was unchanged. These findings indicate that the presence of overt hepatic tumour results in gross derangements of hepatic blood flow. These changes must be taken into consideration when attempting to potentiate the delivery of cytotoxic drugs to hepatic tumour by manipulation of hepatic haemodynamics.

The hepatic perfusion index (HPI), that is the ratio of hepatic arterial to total hepatic blood flow determined by dynamic scintigraphy is elevated in the presence of overt tumour in man (Leveson *et al.*, 1983). Previous studies have suggested that the blood supply to overt liver tumour is derived principally from the hepatic artery (Taylor *et al.*, 1979). Consequently, it has been assumed that the elevations in the HPI in patients with overt hepatic metastases is related to increases in hepatic artery flow *per se*. However, this hypothesis cannot be substantiated in man because of the technical difficulties in accurately measuring the various components of hepatic haemodynamics. Therefore, the aim of this study was to establish the changes in hepatic haemodynamics responsible for alterations in the HPI in rats with overt hepatic tumour derived from the intraportal injection of Walker carcinosarcoma cells.

### Materials and methods

### *Inducation of metastases*

Walker 256 carcinosarcoma cells were grown in suspension in Williams E Medium (Flow Laboratories Ltd., Rickmansworth, UK) supplemented with 5% foetal calf serum at 37°C in a 5% $CO_2$ humidified atmosphere. Ninety male Fisher rats approximately 300 g in weight were anaesthetised with intraperitoneal sodium pentobarbitone (6 mg $100\ g^{-1}$ body weight) and the portal vein exposed through a midline incision. Thirty rats received an intraportal inoculation of $1.6 \times 10^7$ Walker carcinosarcoma cells in a volume of 0.2 ml, over a period of 10 s using a 2FG gauge needle. The needle was held in position in the portal vein for approximately 30 s after completion of the injection to minimise the risk of spillage of cells into the peritoneal cavity. Haemostasis was achieved by compression of the portal vein for 30 s. Another group of 30 rats received an intraportal injection of the same volume of deal cells. These were obtained by growing the cells in the incubator and then placing them in a fridge at 4°C for 4 days. Non-viability was then confirmed using trypan blue exclusion. A further group of 30 rats received an intraportal injection of the same volume of isotonic saline. The abdomen was closed in two layers and the animals allowed to recover. All haemodynamic studies were carried out 3 weeks after the intraportal injection of either viable or dead Walker cells or saline.

### *Radionuclide studies*

Ten rats injected intraportally with either viable Walker cells or the same number of dead Walker cells and ten rats receiving saline were anaesthetised with intraperitoneal sodium pentobarbitone and the right common carotid artery exposed through a midline cervical incision. A 10 cm length 2FG polyethylene cannula was introduced into the carotid artery via an arteriotomy. The cannula was screened into position in the left ventricle using a Siemens Siremobile (Siemens Ltd., Sunbury-on-Thames, UK) image intensifier such that its tip was approximately 1 mm below the aortic valve. Adequate expulsion of a small bolus of injectate by the left ventricle was confirmed by injecting 0.1 ml Sodium Meglumine via the cannula. The rats were then placed under an N.E.8900 Gamma camera (Nuclear Enterprises Ltd., Edinburgh, UK) with a 1 cm pinhole collimator linked to a PDP DEK computer (Digital Equipment Corporation, Maynard, Massachusetts, USA). $^{99m}Tc$ sulphur colloid (0.04 ml) with an activity of 80 MBq was injected rapidly as a bolus into the left ventricle using a 50 µl High Pressure Liquid Chromatography syringe. Images were acquired at three frames per second and stored on hard disc for subsequent analysis. A compositie image of the first five frames of each study was constructed and regions of interest (ROI) were drawn around the left ventricle, right kidney and right lobe of the liver as previously described in detail (Nott *et al.*, 1989).

Time activity curves were then generated from these ROIs and each curve was subjected to a maximum of three quadratic smooths. In first pass studies it is assumed that the bolus of radioisotope reaches the hepatic artery and the renal artery at the same time. Therefore the hepatic arterial phase was taken to begin at the start of the kidney arterial phase and extended to the peak of the kidney time activity curve. The portal phase was taken to begin at the peak of the kidney curve and end at the point of recirculation as previously described in detail (Nott *et al.*, 1989). The HPI was derived from the gradients of the arterial ($G_1$) and portal phases ($G_2$)of the liver perfusion curves using the equation:

$$HPI = \frac{G_1}{G_1 + G_2}$$

*Present address: University Department of Surgery, Glasgow Royal Infirmary, Glasgow, UK.
Received 5 April 1991; and in revised form 30 August 1991.

**Haemodynamic measurements**

*Microsphere technique*

Immediately after the completion of the dynamic scintigraphic studies, hepatic and splanchnic blood flow was determined using the radiolablled microsphere technique (McDevitt & Nies, 1976). Briefly, the right and left femoral arteries were cannulated with 2FG Portex cannula (10 cm in length). The cannula in the left femoral artery was connected to a physiological pressure transducer for the continuous measurement of blood pressure and pulse. The right femoral artery cannula was connected to a Sage 351 (Orien Research Incorporated, Cambridge, Massachusetts, USA) withdrawal pump. A reference blood sample was withdrawn from the femoral artery over a 70 s period. Ten seconds after the start of blood withdrawal, each rat received an intraventricular injection of 60,000–80,000 $^{57}$Co Nen-Trac microspheres (16.4 ± 1 microns) suspended in 0.9% N. Saline with 0.01% tween (New England Nuclear, Stevenage, UK), in a volume of 0.4 ml over 20 s. Prior to the injection the microspheres were sonicated for 10 min and then vibrated on the side of a vortex mixer immediately before injection to ensure complete disaggregation of the spheres.

The animals were killed by an overdose of sodium pentobarbitone, 5 min after microsphere injection, the organs removed, weighed, placed in counting vials, and left in a refrigerator for a minimum of 7 days until the $^{99m}$Technetium had decayed to an insignificant amount. The vials were then placed in a Philips PW 4580 Automatic gamma well counter (Pye Unicam Ltd., Cambridge, UK), and the radioactivity counted. Cardiac output and liver blood flow were determined by the method of McDevitt and Nies, 1976). Portal venous inflow was calculated by adding together the flows to the spleen, pancreas and splanchnic organs. If the blood flow to the right and left kidney differed by more than 10% the distribution of microspheres was not considered to be uniform and the results discarded.

*Corrected hepatic arterial flow*

The development of overt tumour in rats receiving viable tumour cells may underestimate hepatic arterial flow as a result of microspheres passing via arteriovenous shunts from the liver to the lung. The possible source of error was corrected for by using the formula:
Corrected Hepatic Arterial Flow (HAFc) = Liver blood flow (ml min$^{-1}$) + lung blood flow (ml min$^{-1}$) – mean lung blood flow in control rats (ml min$^{-1}$).

*Ratio of tumour to liver arterial blood flow*

After completion of the measurement of total hepatic blood flow by the microsphere method, the normal liver tissue and tumour tissue were separated by careful dissection and weighed. The radioactivity in normal liver and tumour tissue was counted in a Philips well counter to derive a tumour/normal liver (T/L) ratio.

*Electromagnetic flowmetry*

Ten animals injected intraportally with either viable or dead Walker cells or saline were anaesthetised with sodium pentobarbitone and the femoral artery exposed and cannulated with a 10 cm length of Portex tubing. The femoral artery cannula was connected to a pressure transducer for continuous recording of blood pressure. Through a midline incision the duodenal loop was exposed and the portal vein and hepatic artery carefully mobilised. Electromagnetic flow probes of appropriate size (Gould Medical Ltd., Lutterworth, UK) were placed around the portal vein cranial to its junction with the pancreaticoduodenal vein and around the hepatic artery. The flow probes were zeroed *in vivo* by occluding the vessels distal to the probes. Blood flow in both the portal vein and the hepatic artery were recorded using a Gould Flowmeter (Model SP2202, Gould Medical Ltd., Lutterworth, UK).

*Portal pressure*

Immediately after measurement of the portal venous and hepatic artery flows by electromagnetic flowmetry, the probes were removed. The portal vein was cannulated retrogradely using a 2FG paediatric venflon 2 cm below the porta hepatis so that the tip of the cannula lay just above the confluence of the splenic and portal veins. No mobilisation of the liver was required for the procedure and hepatic trauma was avoided during insertion of the cannula.

Splanchnic and portal venous resistances were calculated from the following equations:

$$\text{Spanchnic vascular resistance} = \frac{\text{Mean arterial blood pressure(mmHg)}}{\text{Portal venous flow (ml min}^{-1})}$$

$$\text{Portal vascular resistance} = \frac{\text{Portal pressure (mmHg)}}{\text{Portal venous flow (ml min}^{-1})}$$

*Measurement of intrahepatic arteriovenous shunting*

Intrahepatic shunting was assessed using two methods.

(1) The lung:liver ratio of $^{57}$Co after completion of the microsphere determination of hepatic and splanchnic haemodynamics was used to calculate the degree of intrahepatic arteriorvenous shunting:

$$\text{Arteriovenous shunting} = \frac{\text{Activity in lungs cpm g}^{-1} \times 100}{\text{Activity liver cpm}^{-1}\text{ g}^{-1}}$$

(2) Ten rats injected intraportally with either dead Walker cells or saline were anaesthetised with intraperitoneal sodium pentobarbitone. Through a midline incision the coeliac, hepatic, gastroduodenal and pancreaticoduodenal arteries were exposed.

The distal gastroduodenal artery was ligated with 7/0 silk at its junction with the pancreaticoduodenal artery. A 10 cm long 2 Fr Portex cannula was inserted via an arteriotomy in the gastroduodenal artery and carefully positioned so that its tip lay just distal to the origin of the coeliac and hepatic arteries. A sodium iodide scintillation counter linked to a scalar ratemeter and computer, was placed over the rat's thorax and screened from the abdomen by a lead screen. Under direct vision using an operating microscope 0.05 ml of $^{99m}$Tc methylene diphosphonate ($^{99m}$Tc-MDP) was injected via the hepatic artery cannula over 10 s at physiological pressures (using a high pressure liquid chromatography syringe). The amount of radioactivity passing to the lungs was measured by the scintillation counter. Since $^{99m}$Tc-MDP is not retained within the liver, the majority passes to the lungs and is registered graphically by the computer as a 100% passing fraction. The $^{99m}$Tc-MDP therefore acts as a reference injection. Following administration of the $^{99m}$Tc-MDP, 0.05 ml $^{99m}$Tc-macroaggragated albumen ($^{99m}$Tc-MAA) mean diameter 25 microns (range 15–35 microns) were administered via the cannula using a high pressure liquid chromatography syringe over 10 s. Unlike $^{99m}$Tc-MDP, macroaggregated albumen is trapped within the liver sinusoids and only those particles passing through the liver via arteriovenous shunts appear in the lung field of interest. The percentage passing fraction of $^{99m}$Tc-MAA passing through the liver is represented as a percentage of the passing fraction of the reference injection of $^{99m}$Tc-MDP. Previous studies have indicated an excellent correlation ($r = 0.94$) between this method of measuring intrahepatic arteriovenous shunting and that evaluated by the more classical method using microspheres.

**Statistical anaysis**

Results are expressed as the mean ± s.d. Statistical differences in hepatic haemodynamics between the rats with overt tumour and control animals were evaluated using a Mann Whitney U test.

### Results

### Tumour weight

All rats injected with Walker cells developed overt tumour during the 3 weeks experimental period. However, there was a wide variation in the number (14–26) size, and weight of the tumour between individual rats (6.48 ± 2.44 g, Mean ± s.d. range 1.93–10.11 g). Rats injected with dead Walker cells did not develop any hepatic tumour.

### Hepatic perfusion index

The hepatic perfusion index in control rats was significantly less ($P < 0.01$) than those with overt tumour (Figure 1).

### Changes in hepatic haemodynamics

The hepatic arterial blood flow and the corrected hepatic arterial blood flow were not significantly different between rats with overt tumour and controls measured by either electromagnetic flowmetry or the microsphere technique. In contrast, portal venous inflow (microspheres) and portal flow (electromagnetic flowmetry) were significantly reduced ($P < 0.001$) in rats with overt tumour compared to controls. Although there was no significant difference in cardiac output between the two groups, lung blood flow was significantly increased in rats with overt tumour compared to controls ($P < 0.02$) (Table I).

The reduction in portal venous flow in rats with overt tumour was accompanied by a reduction in portal pressure. Consequently, there was no significant difference in portal vascular resistance between rats with tumour and control rats. Conversely, splanchnic vascular resistance was significantly increased in rats with overt tumour compared to controls (Table II).

**Figure 1** Hepatic Perfusion Index in control rats and animals with overt hepatic tumour. The vertical bars represent the Standard Deviation of the mean.

**Table I** Hepatic artery, portal venous inflow and splanchnic blood flow in controls and animals with overt hepatic tumour using the microsphere method

| | *Saline controls* n = 10 | *Dead cells* n = 10 | *Overt metastases* n = 10 |
|---|---|---|---|
| Hep. artery flow (ml min$^{-1}$) | 3.24±1.64 | 3.19±1.75 | 3.53±1.37 |
| Portal venous inflow (ml min$^{-1}$) | 12.53±3.18 | 12.37±2.97 | 6.71±0.86*** |
| Spleen (ml min$^{-1}$) | 0.93±0.82 | 0.95±0.72 | 0.79±0.39 |
| Pancreas (ml min$^{-1}$) | 1.31±0.95 | 1.29±0.72 | 0.87±0.52** |
| Stomach (ml min$^{-1}$) | 1.59±0.68 | 1.62±0.48 | 0.79±0.36* |
| Small bowel (ml min$^{-1}$) | 6.22±1.86 | 6.23±2.95 | 2.76±0.76*** |
| Large bowel (ml min$^{-1}$) | 3.73±1.11 | 3.41±1.32 | 1.47±0.39*** |
| Lungs (ml min$^{-1}$) | 0.83±0.07 | 0.78±0.14 | 1.18±0.49** |
| Cardiac output (ml min$^{-1}$) | 76.48±5.38 | 77.39±8.37 | 68.51±8.11 |

The results are expressed as mean + 1 Standard Deviation. Asterisks denote a significant difference between the two groups of animals: ***$P < 0.001$; **$P < 0.02$; *$P = 0.05$ (Mann Whitney U test).

**Table II** Hepatic artery and portal vein flow in controls and animals with overt tumour measured using electromagnetic flowmetry

| | *Saline controls* n = 10 | *Dead cells* n = 10 | *Overt metastases* n = 10 |
|---|---|---|---|
| Hepatic artery flow (ml min$^{-1}$) | 3.32±0.42 | 3.21±0.38 | 3.27±0.58 |
| Portal flow (ml min$^{-1}$) | 16.27±1.36 | 16.99±2.11 | 11.88±1.45*** |
| Portal pressure (mmHg) | 8.83±1.13 | 8.87±1.48 | 5.44±2.11** |
| Splanchnic vascular resistance (mmHg ml$^{-1}$ min) | 6.82±0.72 | 6.73±0.46 | 9.59±2.99* |
| Portal venous resistance (mmHg ml$^{-1}$ min) | 0.52±0.16 | 0.57±0.14 | 0.46±0.21 |

The results are expressed as mean + 1 Standard Deviation. Asterisks denote a significant difference between the two groups of animals: ***$P < 0.001$; **$P < 0.02$; *$P + 0.05$ (Mann Whitney U test).

### Tumour:liver ratio

The tumour:liver ratio of cobalt microspheres was 1.18 + 0.18.

### Intrahepatic shunting

Following intraventricular administration of the microspheres there was a significant increase ($P < 0.001$) in the lung:liver ratio of $^{57}$Co in rats with overt tumour (0.36 + 0.11) compared to control rates (0.22 + 0.07) indicative of increased intrahepatic arteriovenous shunting. similarly, the passage of $^{99m}$Tc-MAA was significantly higher in rats with overt tumour than control rats (Figure 2).

### Discussion

The results of this study confirm previous observations in man that the HPI determined by dynamic scintigraphy is altered in the presence of hepatic tumours (Leveson et al., 1985). There was no correlation between the size of the tumours and changes in the HPI. Indeed the elevations in the HPI were of a similar magnitude to those observed in rats with micrometastatic tumour derived from intraportal administration of Walker cells (Nott et al., 1989). Furthermore, our

**Figure 2** Percentage passage of MAA through the liver in rats with overt tumour compared to controls. The vertical bars represent the Standard Deviation of the mean.

results support previous suggestions that the blood supply to hepatic tumour tissue is principally derived from the hepatic artery (Taylor *et al.*, 1979). The increase in the HPI in the presence of overt liver tumour is not however related to the changes in the hepatic arterial flow *per se* as was previously suggested (Leveson *et al.*, 1985), since this was not significantly different between the two groups of control animals and those with tumour. However both portal venous inflow and portal venous flow were significantly decreased in the presence of overt tumour suggesting that these haemodynamic changes are responsible for the alterations in the HPI, at least in the model of hepatic tumour used for this study. However, it remains to be established whether or not alterations in the HPI in patients with liver tumour are related to alterations in hepatic artery or portal venous flows.

In the present study, the presence of overt hepatic tumour was associated with a reduction in portal venous inflow and flow and an increase in splanchnic vascular resistance. The precise mechanism whereby hepatic tumour increases splanchnic vascular resistance is unclear. It is conceivable that the reduction in portal venous flow and portal venous inflow and the increase in splanchnic vascular resistance may result from mechanical compression of the portal triads by the tumour. In this situation, increased intrahepatic mechanical resistance to flow should result in an increase in portal pressure and portal vascular resistance. Indeed, we have previously demonstrated that portal vascular resistance and portal pressure are increased while portal venous flow and inflow are decreased during the early growth and development of hepatic tumour following intraportal administration of Walker cells (Nott *et al.*, 1989). Therefore in the presence of a large tumour mass within the liver it might be expected that there might be more extensive compression of the portal radicles and further elevations in portal pressure and portal vascular resistance. However in the present study, portal pressure was significantly reduced in rats with overt tumour compared to the two groups of control animals while portal vascular resistance was not significantly different between the three groups of animals. Therefore it would appear that any increase in intrahepatic portal resistance resulting from mechanical compression of the portal triads by the tumour must be opposed by changes in hepatic haemodynamics which reduce portal pressure and resistance. A possible explanation for this phenomenon is that portal blood is shunted through and around the tumour by intrahepatic vascular pathways and by the development of an extrahepatic circulation which maintains portal vascular resistance at normal levels and reduces portal pressure. In cirrhosis, the portal pressure remains elevated despite the development of an extensive collateral circulation because of the accompanying hyperdynamic state, namely an increase in cardiac output and increased splanchnic blood flow. In the present study however, overt hepatic tumour does not induce a hyperdynamic state since cardiac output was unchanged and portal venous flow and portal venous inflow were decreased. Furthermore, portal pressure is significantly decreased in the presence of overt tumour whilst splanchnic vascular resistance is increased. These observations therefore suggest that the presence of overt tumour in rats is associated with an increase splanchnic vasoconstriction. The mechanisms whereby overt hepatic tumour elicits splanchnic vasoconstriction are not clear but may result from release of vasoactive substances. Indeed, our own recent studies have confirmed the release of vasoactive substances during the growth and development of hepatic micrometastases (unpublished).

The presence of overt hepatic tumour in the rat was accompanied by a marked increase in arteriovenous shunting as evidenced by an increased passage of $^{57}$Co and $^{99m}$Tc-MAA through the liver. These observations are therefore in accord with a previous observation which also indicated the presence of intrahepatic arteriovenous shunts assoicated with liver metastases derived from colorectal carcinoma in man (Starkhammer *et al.*, 1987). The precise nature of these intrahepatic arteriovenous anastomoses is not known, but they could arise from the 'opening up' of pre-existing shunts. Alternatively shunts could develop *de novo* within or around the tumour. Nevertheless, the existance of arteriovenous shunts in the presence of overt tumour may explain why regional chemotherapy via the hepatic artery appears to have a marginal therapeutic effect, since a large proportion of the cytotoxic may be shunted around or through the tumour.

In summary therefore, the results of this study indicate that overt tumour derived from the intraportal inoculation of Walker cells results in an increase in the HPI. The blood supply to the tumour is derived principally from the hepatic artery. However hepatic arterial flow does not change in the presence of tumour and the alterations in the HPI are secondary to a reduction in portal venous inflow. Moreover, the presence of overt hepatic tumour is associated with gross derangement of hepatic haemodynamic with a pronounced increase in intrahepatic arteriovenous shunting. The haemodynamic changes accompanying the development of overt hepatic tumour are complex and must be taken into account when attempting to potentiate the distribution of cytotoxics to the tumour by regional administration or through manipulation of liver blood flow.

### References

McDEVITT, D.G. & NIES, A.S. (1976). Simultaneous measurement of cardiac output and it's distribution with microspheres in the rat. *Cardiovasc. Res.*, **10**, 494.

LEVESON, S.H., WIGGINS, P.A., NASIRU, T.A., GILES, G.R., ROBINSON, P.J. & PARKIN, A. (1983). Improving the detection of hepatic metastases by the use of dynamic scintigraphy. *Br. J. Cancer*, **47**, 719.

LEVESON, S.H., WIGGINS, P.A., GILES, G.R., PARKIN, A. & ROBINSON, P.J. (1985). Deranged liver blood flow patterns in the detection of liver metastases. *Br. J. Surg.*, **72**, 128.,

NOTT, D.M., GRIME, J.S., YATES, J., DAY, D.W., BAXTER, J.N., JENKINS, S.A. & COOKE, T.G. (1989). Changes in the hepatic perfusion index during the development of experimental hepatic tumours. *Br. J. Surg.*, **76**, 259.

TAYLOR, I., BENNETT, R. & SHERRIF, S. (1979). The blood supply of colorectal liver metastases. *Br. J. Cancer*, **39**, 749.

STARKHAMMAR, H., HAKANSSON, L., MORALES, O. & SVEDBERG, J. (1987). Effect of microspheres in intra-arterial chemotherapy. A study of arterio-venous shunting and passage of a labelled marker. *Med. Oncol. Tum. Pharmacother.*, **4**, 87.


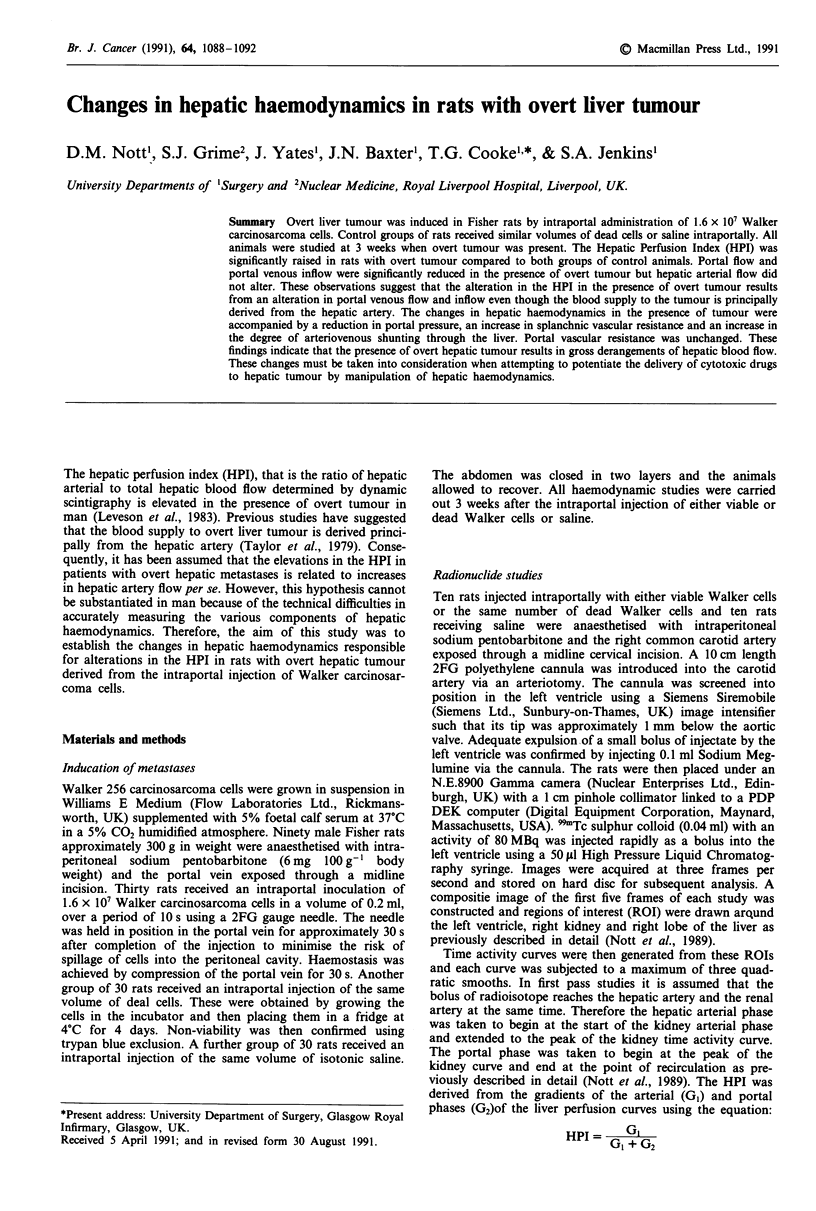

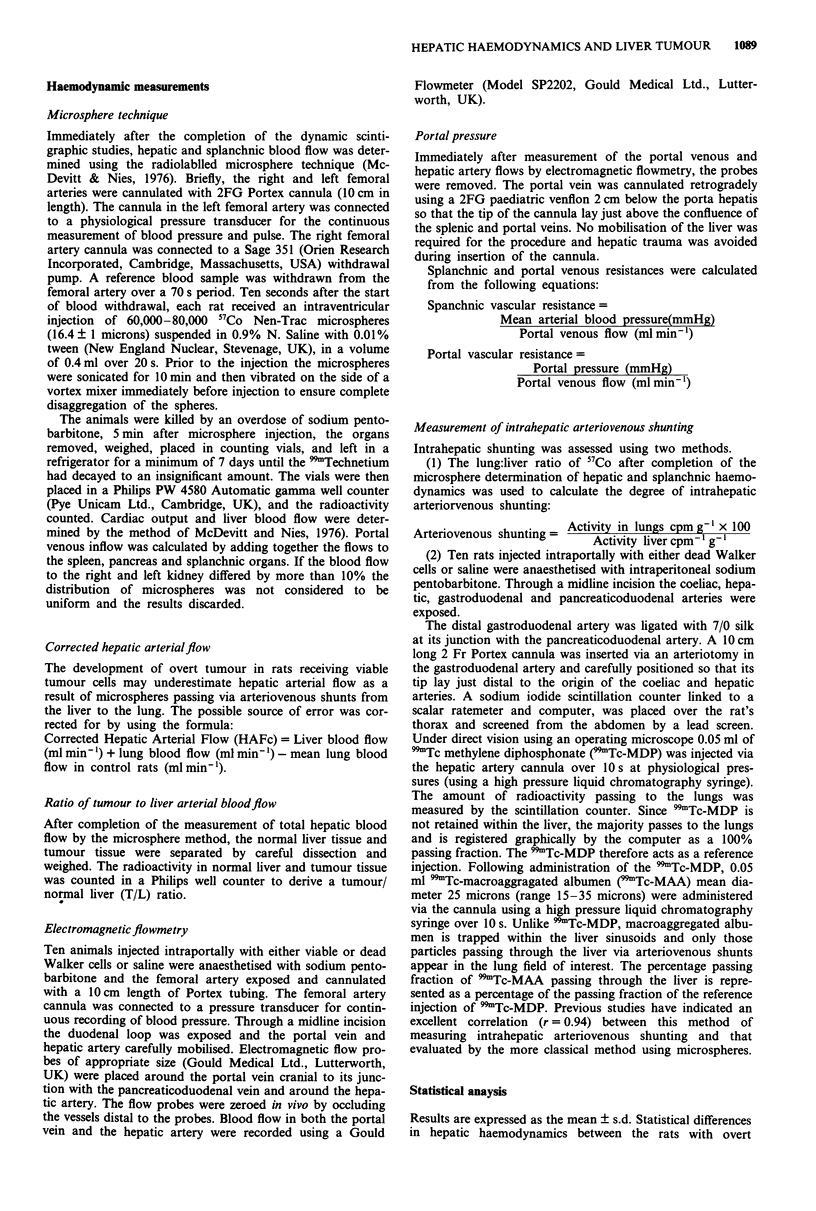

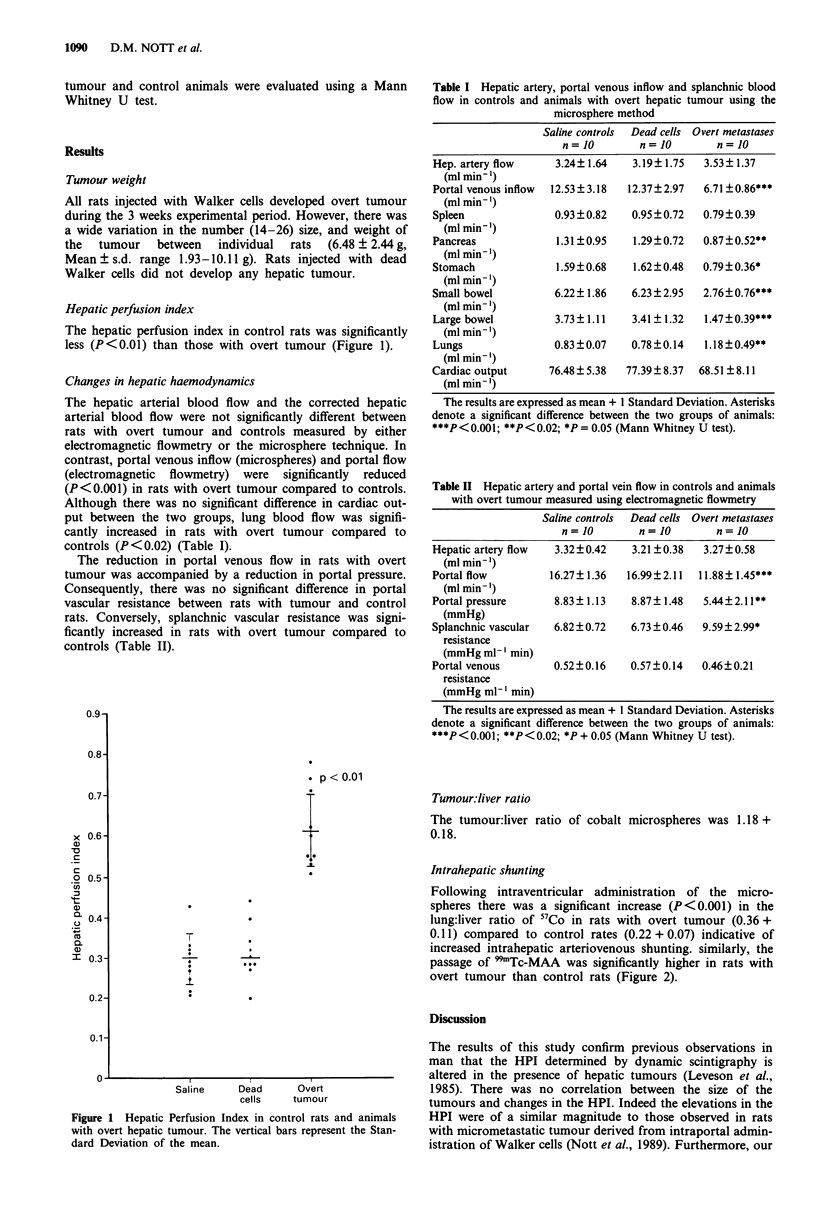

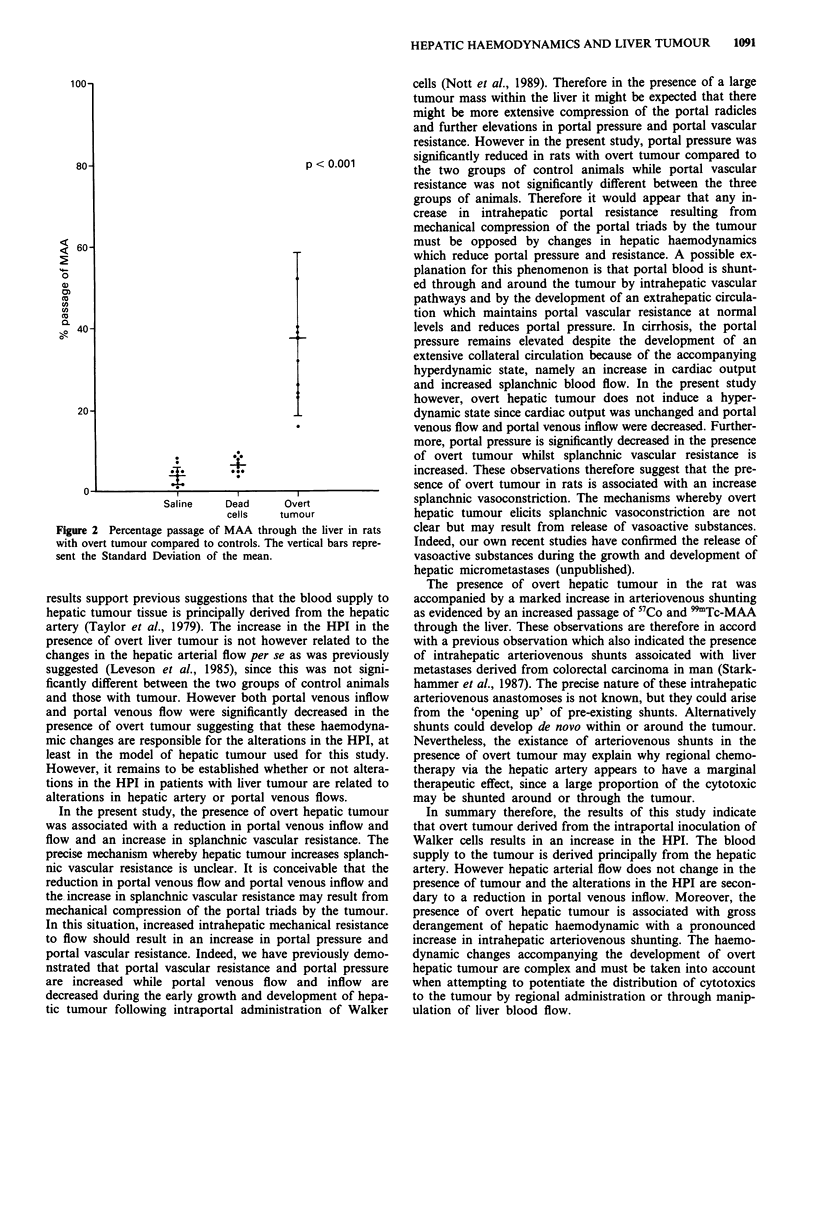

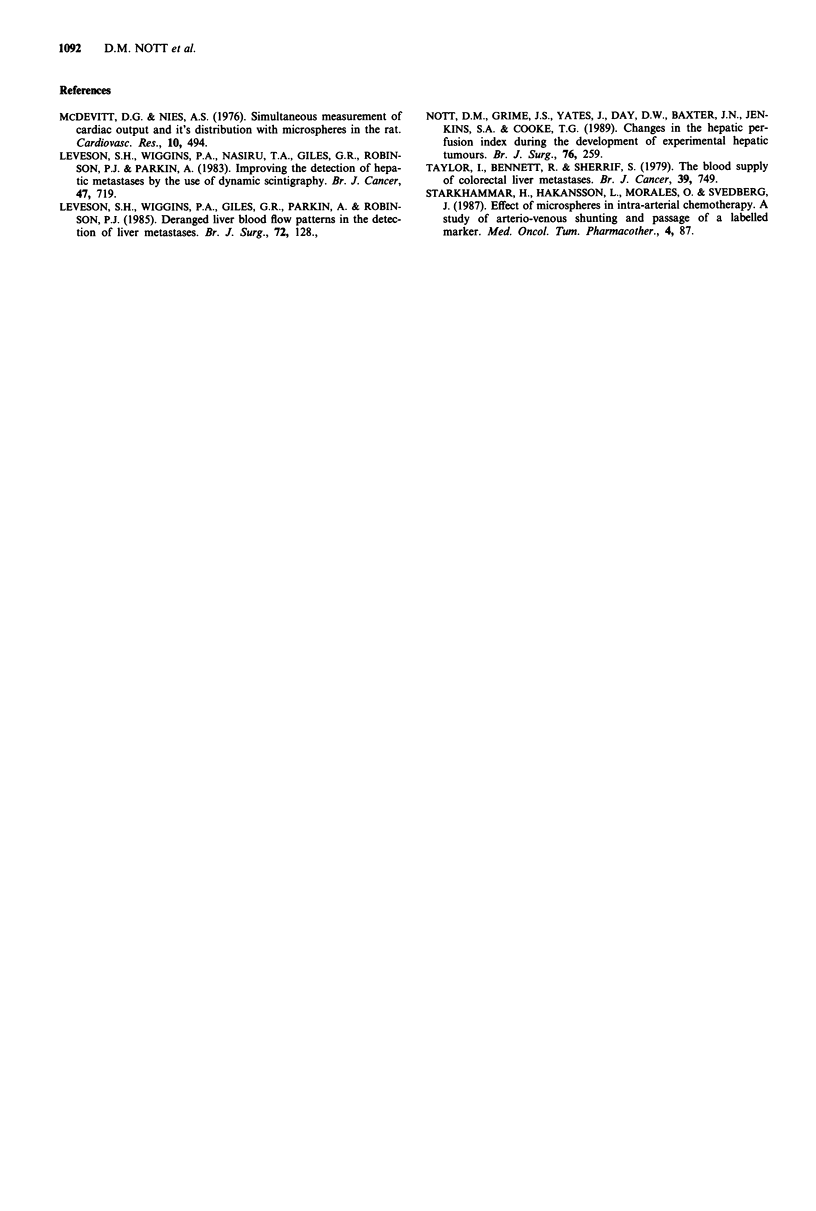


## References

[OCR_00665] Leveson S. H., Wiggins P. A., Giles G. R., Parkin A., Robinson P. J. (1985). Deranged liver blood flow patterns in the detection of liver metastases.. Br J Surg.

[OCR_00661] Leveson S. H., Wiggins P. A., Nasiru T. A., Giles G. R., Robinson P. J., Parkin A. (1983). Improving the detection of hepatic metastases by the use of dynamic flow scintigraphy.. Br J Cancer.

[OCR_00654] McDevitt D. G., Nies A. S. (1976). Simultaneous measurement of cardiac output and its distribution with microspheres in the rat.. Cardiovasc Res.

[OCR_00672] Nott D. M., Grime S. J., Yates J., Day D. W., Baxter J. N., Jenkins S. A., Cooke T. G. (1989). Changes in the hepatic perfusion index during the development of experimental hepatic tumours.. Br J Surg.

[OCR_00680] Starkhammar H., Håkansson L., Morales O., Svedberg J. (1987). Effect of microspheres in intra-arterial chemotherapy. A study of arterio-venous shunting and passage of a labelled marker.. Med Oncol Tumor Pharmacother.

